# Fluorogenic Hyaluronan Nanogels Track Individual Early Protein Aggregates Originated under Oxidative Stress

**DOI:** 10.1021/acsami.3c13202

**Published:** 2024-01-09

**Authors:** Matteo Cingolani, Francesca Lugli, Mirko Zaffagnini, Damiano Genovese

**Affiliations:** † Dipartimento di Chimica “Giacomo Ciamician”, Università di Bologna, 40126 Bologna, Italy; ‡ Dipartimento di Farmacia e Biotecnologie, Università di Bologna, 40126 Bologna, Italy

**Keywords:** protein aggregation, early diagnosis, fluorogenic probe, fluorescence microscopy, nanotracking, fluorescence correlation spectroscopy (FCS), hyaluronic acid, glycosaminoglycan (GAG), glyceraldehyde-3-phosphate dehydrogenase (GAPDH)

## Abstract

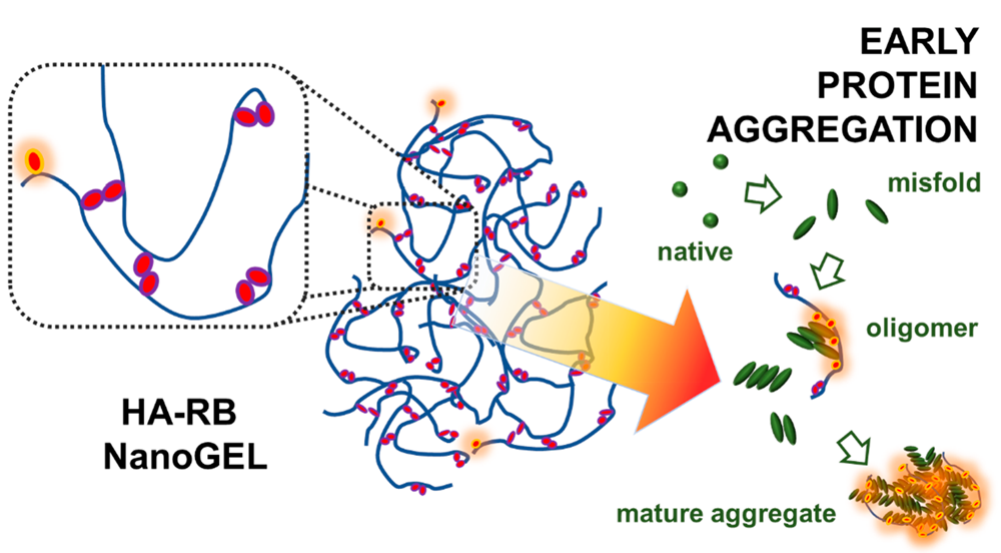

Proteins are broadly versatile biochemical materials, whose functionality is tightly related to their folding state. Native folding can be lost to yield misfolded conformations, often leading to formation of protein oligomers, aggregates, and biomolecular phase condensates. The fluorogenic hyaluronan HA-RB, a nonsulfonated glycosaminoglycan with a combination of polyanionic character and of hydrophobic spots due to rhodamine B dyes, binds to early aggregates of the model protein cytoplasmic glyceraldehyde-3-phosphate dehydrogenase 1 from *Arabidopsis thaliana* (AtGAPC1) since the very onset of the oligomeric phase, making them brightly fluorescent. This initial step of aggregation has, until now, remained elusive with other fluorescence- or scattering-based techniques. The information gathered from nanotracking (via light-sheet fluorescence microscopy) and from FCS in a confocal microscope converges to highlight the ability of HA-RB to bind protein aggregates from the very early steps of aggregation and with high affinity. Altogether, this fluorescence-based approach allows one to monitor and track individual early AtGAPC1 aggregates in the size range from 10 to 100 nm with high time (∼10–2 s) and space (∼250 nm) resolution.

## Introduction

Among all possible conformations of proteins, their native fold is thermodynamically favored; yet the activation barrier toward misfolded conformations—often the first step toward formation of protein oligomers, aggregates, and condensates—can be overcome via physicochemical transformations triggered by changes in pH, temperature, or by the action of oxidative stress, crowding effects, or interactions with other biomolecules such as nucleic acids^1,2^ and glycosaminoglycans (GAGs). GAGs, for instance, have been broadly recognized as ligands for prions and the protein α-synuclein and, depending on their backbone structure, charge density and degree of sulfation, are involved in modulating or even triggering the aggregation process.^3−5^

Monitoring early stage processes in protein aggregation is essential for the effective understanding and tackling of related neurodegenerative diseases, diabetes, and of other biological processes arising from protein misfolding leading to aggregation.^6,7^ Beside the archetype thioflavin-T (ThT), other fluorescent dyes,^8,9^ aggregation-induced-emission fluorogenic moieties (AIEgens),^10−13^ and luminescent conjugated oligomers and polymers (LCOs and LCPs)^14,15^ have been proposed for monitoring the early stages of protein aggregation.^16^ Among these novel probes, LCOs and LCPs stand out due to the versatility of their structure, tailored to optimize the interactions with protein aggregates, with the most performing ones displaying a polyanionic character (multiple negatively charged carboxylates and/or sulfonates) and a hydrophobic backbone, yielding only partial water solubility. This versatility is difficult—if not impossible—to obtain with molecular probes, depending on their specific chemical structure. In the most interesting probes, the luminescence is quenched in water and switches on (in some cases also shifting in wavelength) when the probes interact with protein aggregates, thus yielding a fluorogenic (and eventually ratiometric) probe.^17,18^

Here, we introduce a nonsulfonated GAG—hyaluronan—functionalized with rhodamine B (HA-RB), which forms nanogels that are self-quenched in water and are able to interact with specific surfaces according to their hydrophobicity and softness, switching on the photophysics of the RB dyes.^19^ We investigate the potential of HA-RB to bind and be brightly fluorescent the early stage protein aggregates. As a model protein, we chose cytoplasmic glyceraldehyde-3-phosphate dehydrogenase 1 from *Arabidopsis thaliana* (AtGAPC1) because it has recently been demonstrated to form insoluble and globular aggregates following the formation of a mixed disulfide between its catalytic cysteine and a molecule of glutathione (i.e., S-glutathionylation).^20^ We focused on the formation of fluorescent species close to—or within—the aggregation lag time of AtGAPC1 with a variety of fluorescence techniques, including fluorescence assays, confocal microscopy, FCS, and light-sheet microscopy for nanotracking.

## Materials and Methods

### AtGAPC1 Redox-Driven Aggregation

AtGAPC1 was expressed and purified as previously described.^21^ Recombinant protein (5 μM) was treated with 0.125 mM H_2_O_2_ supplemented with 0.625 mM GSH in the presence or absence of 200 mM NaCl. All incubations were carried out at room temperature in 50 mM Tris-HCl (pH 7.5), 1 mM EDTA, and 0.14 mM NAD^+^ (buffer A). For fluorescence measurements, ThT and HA-RB were added to the different incubation mixtures at a final concentration of 25 μM and 100 nM, respectively. Control experiments were carried out by incubating AtGAPC1 in buffer A supplemented or not with ThT or HA-RB at concentrations 10, 100, and 1000 nM. At the indicated times, an aliquot was withdrawn to assay residual GAPDH activity as previously described.^22^ Activity data were expressed as a percentage of the maximal control activity. For recovery assays, protein samples were incubated at different time points with 10 mM tris(2-carboxyethyl)phosphine (TCEP). After 15 min of incubation, aliquots were withdrawn for the assay of GAPDH activity. Aggregation kinetics was monitored by measuring the turbidity at 405 nm and the hydrodynamic diameters using dynamic light scattering (DLS) analysis as previously described.^20^

### Dual Fluorescence and DLS Monitoring of Aggregation Kinetics

Aggregation of AtGAPC1 was monitored with a dual spectrofluorimetric and scattering assay, using either ThT (25 μM) or HA-RB (100 nM) as the fluorescent probe in a Tris/EDTA pH 7.4 buffer solution containing protein (5 μM) and NAD (0.14 mM). This solution was split in two which were used to monitor fluorescence and scattering simultaneously at 25 °C. The fluorescence and scattering signals were first monitored for 15 min: in this time frame nothing was observed, because the aggregation process is initiated by the addition of the oxidative stress factors H_2_O_2_ (0.125 mM) and GSH (0.625 mM). After their addition, fluorescence and DLS are monitored for 100 min, when the aggregation kinetics reaches its plateau. All experiments have been performed at a controlled temperature of 25 °C. In the experiments involving the tuning of the lag phase, it has also been added to the solution NaCl at a concentration of 200 mM before adding the oxidative stress factors.

### Fluorogenic Probe HA-RB: Synthesis and Characterization

The fluorogenic functionalization of hyaluronic acid (HA-RB) was obtained from a previous synthetic method.^19^ Briefly, HA (200–600 kDa) was covalently functionalized with Rhodamine B isothiocyanate (RITC) in DMSO and then purified with dialysis (cutoff 12 kDa), yielding a dispersion of functionalized HA-RB in aqueous solution (PBS pH 7.4) with doping degree of 1 RB moiety every ca. 60 HA monomers (1.8% doping degree of HA monomer units, i.e., ∼18 RB dyes per HA polymer chain). HA-RB has an intrinsic tendency to form aggregates in water, driven by the rigidity of this polyanion and by the presence of RB moieties—with their hydrophobic contribution. DLS yields a hydrodynamic diameter of these aggregates in the 100–300 nm size range. The very low scattering and sedimentation observed indicate a strong hydration of the aggregates which are therefore identified as nanogels.^19^ RB moieties in the HA-RB nanogels in water suffer from heavy self-quenching, displaying an average PLQY of 0.0012, which results in a low fluorescence background in microscopy-based experiments.

## Results

**Figure 1:**
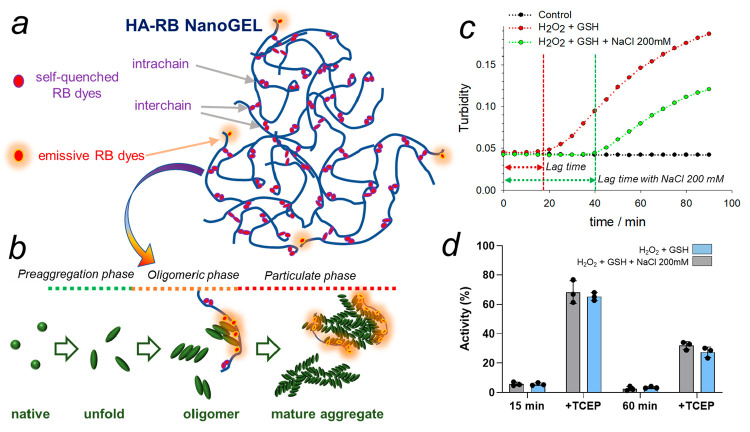
(a) Cartoon of the HA-RB nanogels, formed by hyaluronan chains (blue) functionalized by RB dyes that are massively self-quenched in water (purple-red ovals) with only <1% of the dyes in an emissive state (orange-red ovals). (b) Cartoon of protein aggregation main steps and of the interaction of HA-RB nanogels with oligomeric or mature aggregates, which turns on the bright fluorescence of RB dyes. (c) Aggregation kinetics of AtGAPC1 with a redox trigger (H_2_O_2_ and GSH, red line and dots) and with a redox trigger in the presence of 200 mM NaCl (green line and dots). The control is the same protein solution in the presence of 200 mM NaCl mM without a redox trigger (black line and dots). (d) Residual activity and recovery of AtGAPC1 after 15 and 60 min with the redox trigger (H_2_O_2_ and GSH) in the absence (blue boxes) or presence of 200 mM NaCl (gray boxes).

In a previous study, we stated that the redox-dependent aggregation process of AtGAPC1 follows three phases.^20^ In the first phase (i.e., preaggregation phase), the enzyme loses its functionality without significantly changing its tetrameric conformation. In addition, enzyme activity can partially be restored by reducing treatments. The initial hydrodynamic diameter (dH) remains the one measured for soluble AtGAPC1 dH = 9.2 ± 0.5 nm, a value compatible with the crystal structure of AtGAPC1 tetramers.^22^ AtGAPC1 could thus stay glutathionylated for 5 min without significantly changing its overall native conformation. True aggregation starts only later, during the “oligomeric phase”, when AtGAPC1 gradually accumulates in a permanent inactive state with formation of insoluble aggregates, and the recovery of AtGAPC1 activity becomes less and less efficient, indicating that oligomerization is associated with a permanently inactivated state of the protein. During this lag phase (preaggregation and oligomeric phases, overall ca. 15–20 min), AtGAPC1 aggregates are still rather small (d_H_ < 100 nm) and are only barely visible with light scattering techniques, with very low increase in overall counts.^20^ Then, 15–20 min after initiating the oxidative stress, protein aggregates abruptly start to grow in number and size in the third phase (the “particulate” phase), with DLS yielding d_H_ starting at ∼100 nm to finally reach micrometric dimensions. Final aggregates appear to be made by the random binding of nearly globular particles from ∼300 to 500 nm in d_H_, linked together to form irregularly branched chains, with no formation of fibrils. The described phases are schematically indicated in Figure 1b.

Favorable interactions of HA-RB nanogels toward soft and hydrophobic nanosurfaces were previously described, with a high affinity of this probe demonstrated toward PEGylated silica nanoparticles^19^ and nanoplastics.^23^ Starting from this previous knowledge, we tested the ability of HA-RB to probe the aggregation process of AtGAPC1 with particular attention to early stage aggregates.

### Monitoring the Aggregation of AtGAPC1 with the Fluorogenic Probe HA-RB

**Figure 2:**
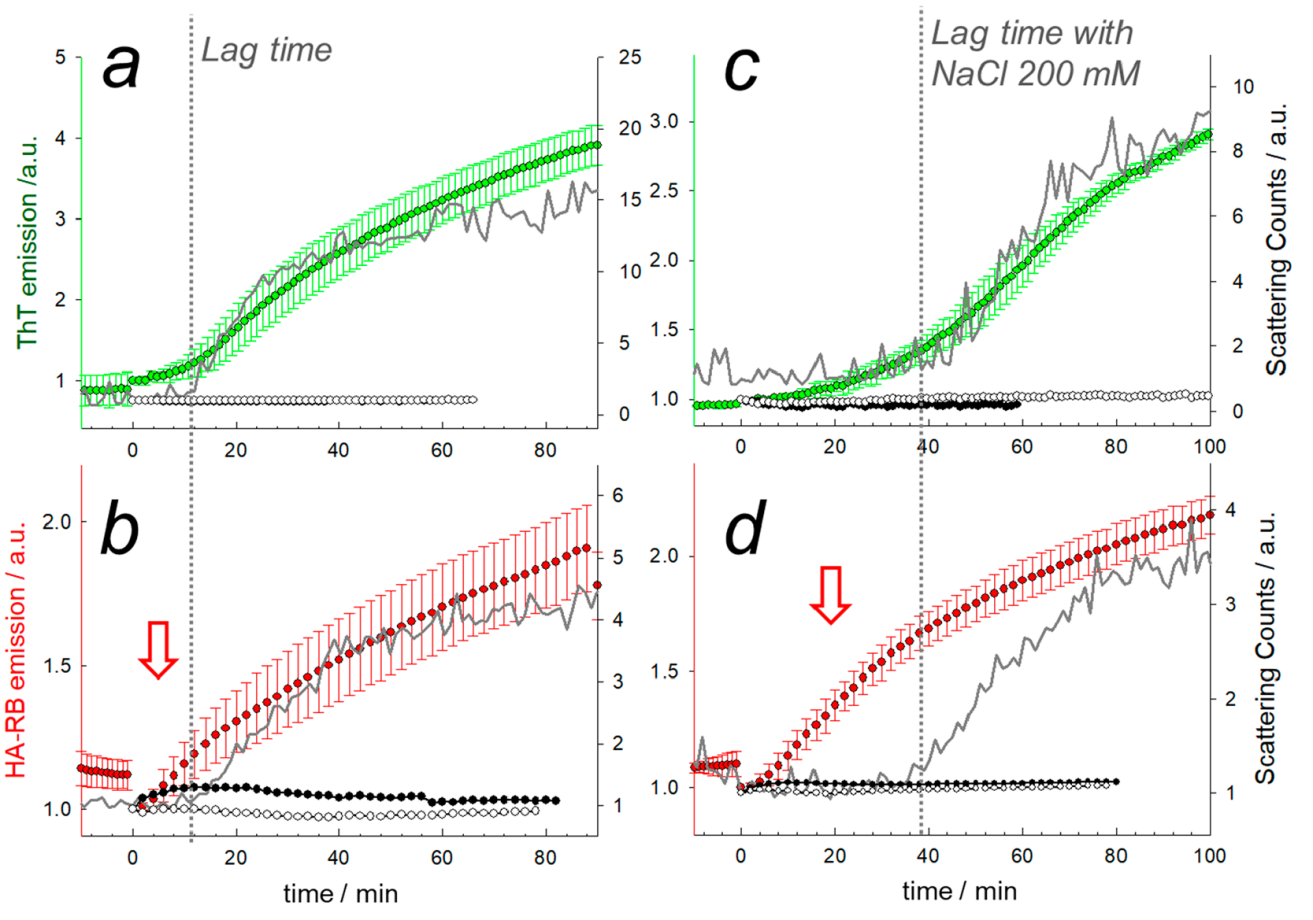
Fluorescence assays during the aggregation process in cuvettes with ThT or HA-RB without (a, b) and with (c, d) the addition of 200 mM NaCl. Green lines and dots: fluorescence trend at 480 nm of ThT (excitation wavelength 400 nm). Red lines and dots: fluorescence trend at 580 nm of HA-RB (excitation wavelength 530 nm). Gray lines: scattering counts from DLS. Black and white circles: control experiments in absence of protein or of GSH, respectively. Red arrows highlight the early enhancement of fluorescence observed with the fluorogenic probe HA-RB during the lag phase.

In the presence of the fluorogenic probe HA-RB at concentration 100 nM, the aggregation kinetics of oxidized AtGAPC1 (i.e., S-glutathionylated AtGAPC1) is not altered, as measured by turbidity and DLS (Figure 2). In addition, HA-RB has no effect on the functionality of the enzyme, which maintains the same activity measured at different times under control conditions (98 ± 4% of control activity). We then proceed by monitoring the fluorescence emission of HA-RB, at concentration 100 nM, during aggregation in parallel with DLS analysis. Triplicate measurements show a reproducible trend in which fluorescence starts to grow only a few minutes after the beginning of the redox treatment, while DLS confirms that pronounced scattering enhancement only takes place after the lag phase of ∼15 min (Figure 2b). The overall enhancement appears limited, reaching an average 250% signal enhancement, yet it pairs the enhancement provided by ThT for these nonfibrillar aggregates (Figure 2a and b). Furthermore, the real local enhancement of emission on the surface of aggregates might be much higher since HA-RB localized on the aggregates likely displays higher PLQY than measured in the average solution.

The fluorescence signal of HA-RB, different from that of ThT, shows a very early enhancement, and it does not appear to follow a lag phase, as instead shown by the emission of ThT which is similar to the one detected via scattering-based methods (Figure 2a and b). This peculiar early enhancement, during the lag phase, bears potential for further investigations. Interestingly, we find out that the presence of NaCl (200 mM) is an easy handle to tune the duration of the lag phase, with a significant increase of the lag time from ∼15 to ∼40 min (Figure 1c). Despite an altered lag phase, the presence of NaCl does not affect the sensitivity of AtGAPC1 to S-glutathionylation, suggesting that the redox switch of the catalytic cysteine is maintained at high ionic strength (Figure 1d). Indeed, the enzyme maintains the same residual activities after exposure to H_2_O_2_ and GSH (15 and 60 min) in the presence or absence of a high NaCl concentration. In addition, the recovery from inactivation by reductants (TCEP) is not influenced by the presence of salt and thus again similar in the absence or presence of high NaCl concentration. Finally, also the morphology of final aggregates is indistinguishable whether they are grown in the presence of NaCl 200 mM or not.

Based on these findings, we decided to investigate the initial steps of the aggregation kinetics in the presence of high ionic strength (200 mM NaCl), with the aim of prolonging the lag phase and to gain more detail on the preaggregation events. The fluorescence assays with HA-RB and ThT were repeated in the presence of high NaCl concentration, simultaneously with DLS monitoring. We observed, similarly to the previous experiments, that HA-RB shows a distinct fluorescence intensity increase at a much earlier stage compared to ThT and to DLS counts, confirming that, even at high NaCl concentration, HA-RB has a high affinity for early aggregates and that it shows enhanced fluorescence quantum yield of the initially self-quenched RB moieties (initial PLQY is 0.0012). It is important to note that HA-RB does not show any intensity drift in the experimental conditions, even in the presence of high NaCl concentration, and the redox chemical triggers H_2_O_2_ and GSH, or in the presence of the protein without the redox chemical triggers (control curves in Figure 2). The same difference in the emission onset during aggregation is observed

This clear difference in the intensity trend at the beginning of the aggregation process represents an important preliminary assessment of the potential of HA-RB as a probe for early stage aggregation.

### Fluorescence Microscopy: Laser Scanning Confocal Microscopy

Following the promising observations of the bulk experiments in a cuvette, we decided to turn to fluorescence microscopy-based methods with the aim to gain deeper insight into the aggregation process and on the potential of the fluorogenic hyaluronan HA-RB as a diagnostic tool for early stage aggregation.

**Figure 3:**
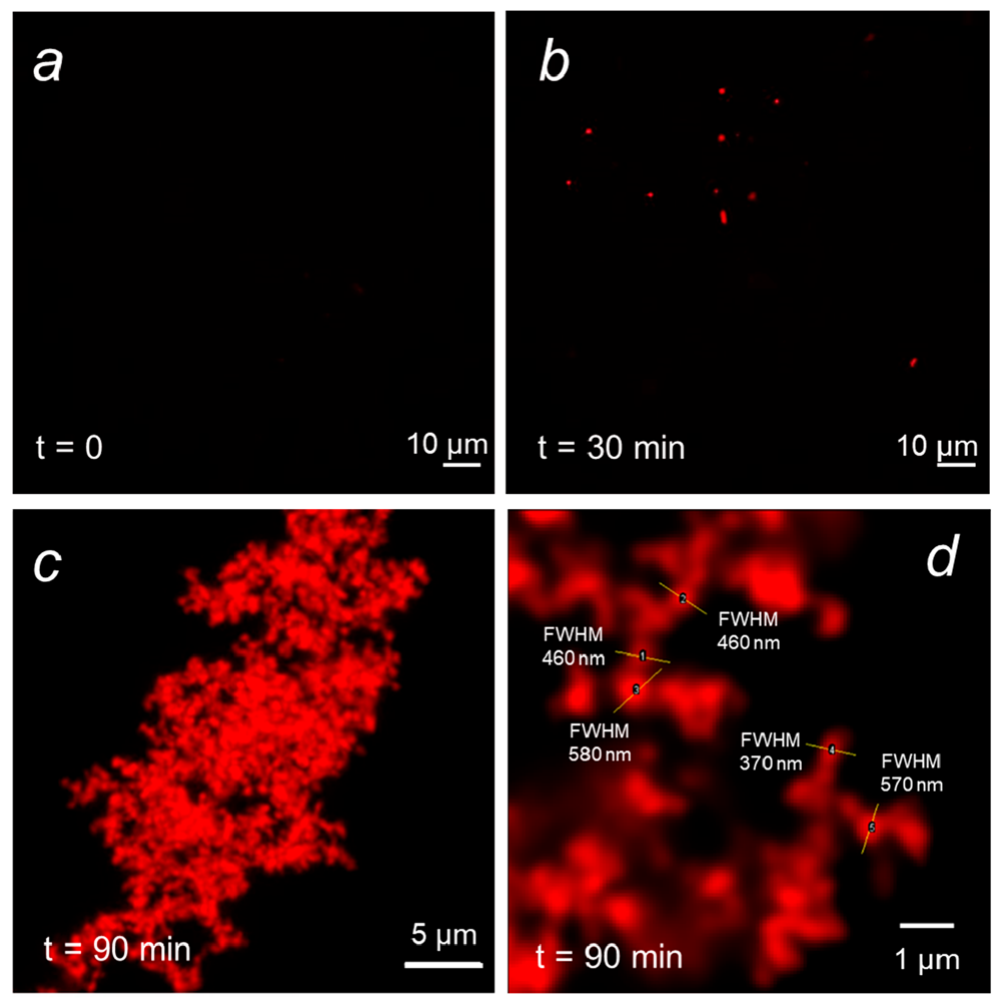
Confocal microscopy images of initial steps of the aggregation process at the time of the redox trigger (a, *t* = 0) and after ∼30 min (b) and of mature aggregates (*t* ∼ 90 min after the redox trigger) labeled with HA-RB. Aggregates diffusing in solution are not visible at the initial step, while they can be observed in the oligomeric phase (b). (c, d) Large conglomerates of particulate aggregates sedimented on the coverglass surface are observed at the end of the aggregation process (90 min). Closeup of the conglomerate (d) showing the typical size of particulate composing the conglomerate, in the range ∼350–600 nm (resolution limit 290 nm).

Confocal microscopy allowed us to acquire detailed micrographs of the aggregates at different stages of their formation (Figure 3). Remarkably, the field of view at the beginning of the aggregation process appears to be homogeneously dark, proving that the initial state of HA-RB nanogels is effectively deeply quenched. The HA-RB nanogels, therefore, do not interfere with the luminescence signal localized on the aggregation hotspots. From 20 to 35 min after triggering the aggregation process, we observe emissive spots below the diffraction limit of the microscope (∼250 nm) or slightly larger (Figure 3b). After ∼40 min, in the presence of high [NaCl], we start to observe a relevant number of emissive spots with size above the diffraction limit, and soon this type of emissive aggregates starts to coalesce, finally reaching the shape of the conglomerates that were previously observed and described with electron microscopy methods^20^ (Figure 3c and d). These images are extracted from live acquisition video at different time steps of aggregation kinetics. At early steps, in particular, misfolded protein, oligomers, and preaggregates diffuse rapidly in solution and cannot be efficiently tracked with our confocal setup in the conditions of acquisition (3.8 fps, 512 × 512 pixels, pixel size 0.25 μm). Our fastest confocal scan did not provide images of sufficient quality for efficient nanotracking of fluorescent aggregates; hence, we could not gain information on early aggregates below the resolution limit via their diffusion properties.

### Nanotracking with Laser-Sheet Wide-Field Microscopy

**Figure 4:**
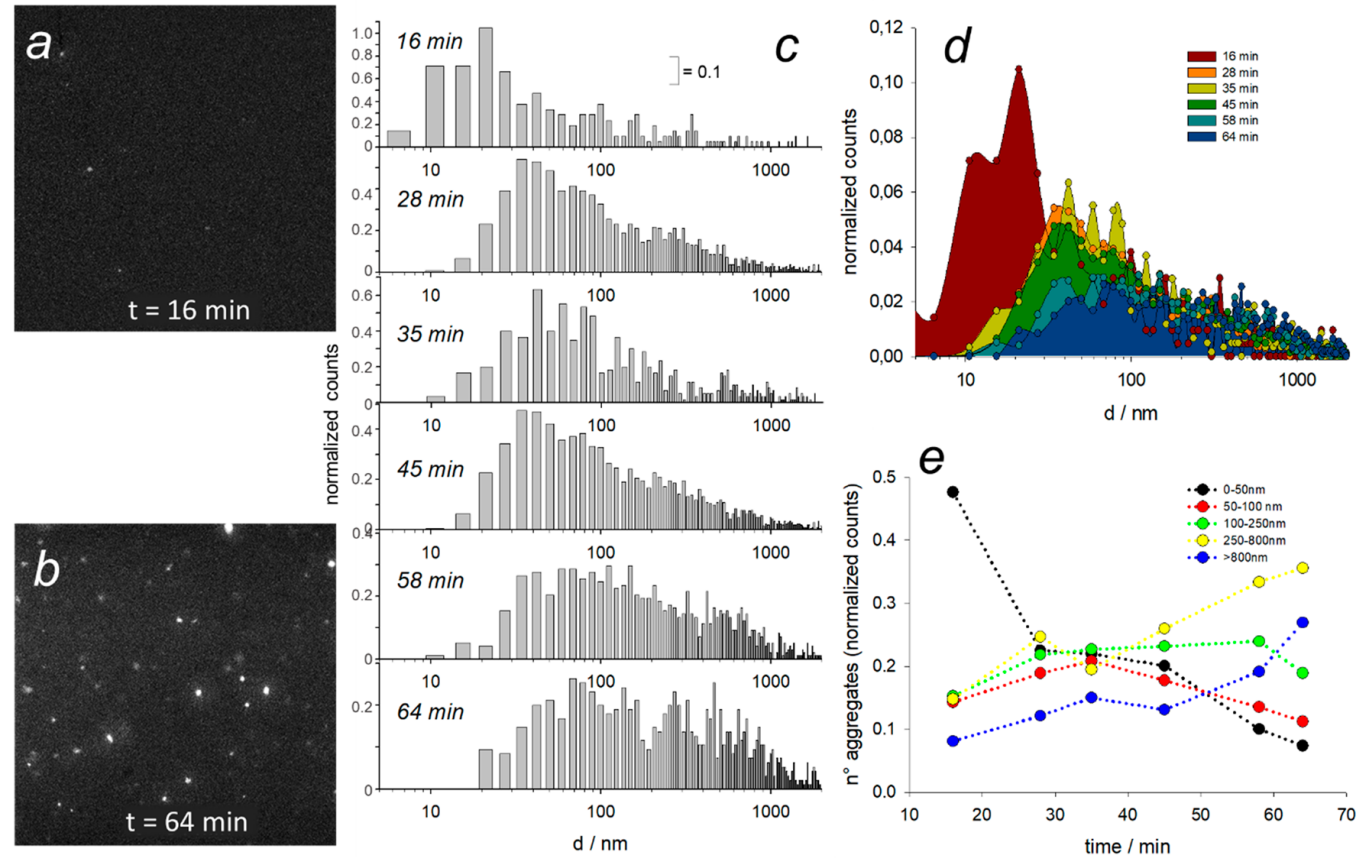
Results of nanotracking from light-sheet microscopy. Representative frames extracted from videos acquired at 16 min (a) and 64 min after redox trigger (lateral frame size = 590 μm). (c) Size distributions calculated from single particle trajectories of video acquisitions at different time steps from 16 to 64 min after trigger. Diameter axis is in logarithmic scale; normalized counts axis has 0.1 division. The same histograms of (c) are also plotted together and overlapped in (d). (e) Trends of populations vs time in the ranges 0–50 nm (black), 50–100 nm (red), 100–250 nm (green), 250–800 nm (yellow), and larger than 800 nm (blue). “*n*° aggregates” denotes the number of aggregates counted by the nanotracking algorithm.

To obtain information on the size of early aggregates below the resolution limit of fluorescence microscopy, we decided to move to a wide-field fluorescence microscope that we modified with a laser-sheet excitation for out-of-focus fluorescence removal (detailed instrumental description in Supporting Information). Laser-sheet microscopy is a well-established technique for imaging and analysis of large volume samples, and it was recently applied in commercially available instrumentation to size analysis of nanometric diffusing materials. With this optical setup, we gained high resolution in time (high S/N images with short acquisition time *t*_acq_ = 35 ms), and we matched spatial resolution to the diffusion of aggregates rather than to their size (10× objective, NA = 0.8, pixel size = 1150 nm). In these conditions, we could acquire short videos (1000 frames, ∼35 s) distributed during the extended lag phase in the presence of a high concentration of NaCl, using a small volume quartz cuvette (500 μL) at controlled temperature *T* = 25 °C (Figure 4a and b). During the lag phase, we aimed thus to track the diffusing preaggregates at the early steps of their formation. We can confirm that the smallest size range (10–50 nm) appears at the beginning of the lag phase (15–20 min from the redox trigger) and almost disappears when the particulate phase takes place (Figure 4c–e). Larger preaggregates below 100 nm in diameter (50–100 nm range) are observed to increase in number during the lag phase and then decrease during the particulate phase, confirming the previous hints from DLS measurements. The middle size (100–250 nm) is present throughout the whole aggregation process, while globular particles that are finally found to compose the large, branched aggregates (250–800 nm) tend to increase also after the lag phase. Finally, consistently with other observations, the very large aggregates (>800 nm) are not present at the beginning and accumulate in the particulate phase (Figure 4c–e). Even though a certain noise must be taken into account in the analysis that leads to the histograms herein shown and discussed, we can also safely state that the composition of the aggregates is extremely polydisperse, with aggregates ranging from <100 nm to micrometers in most steps of aggregation.

### Monitoring Diffusion of Early Aggregates with Fluorescence Correlation Spectroscopy (FCS)

**Figure 5:**
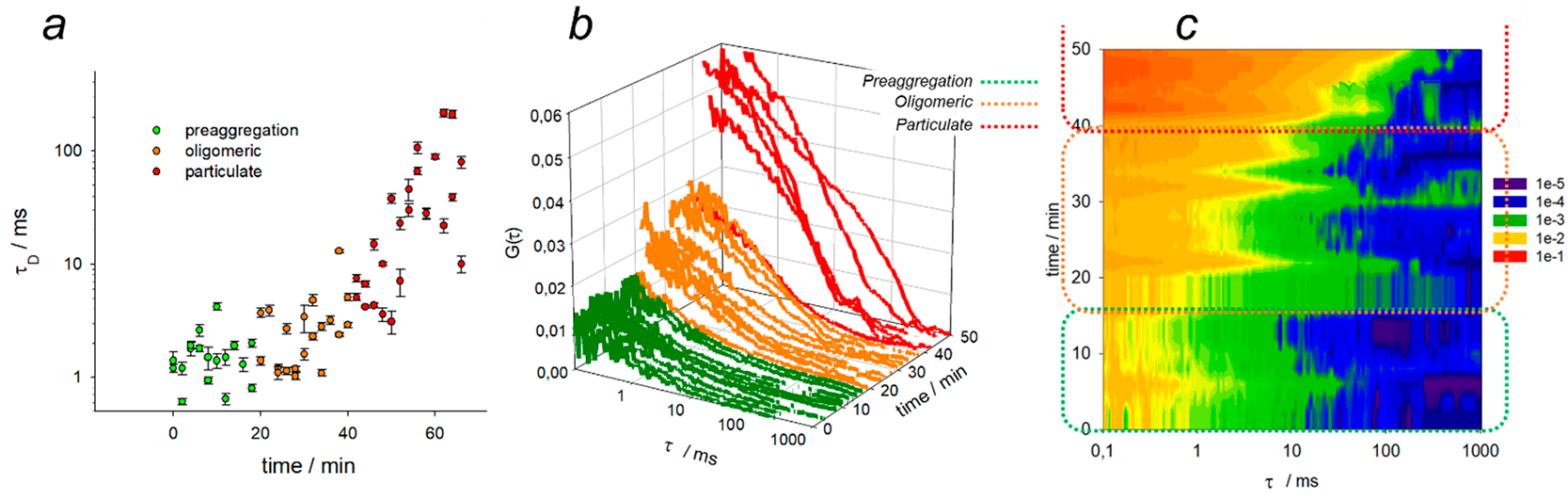
Results of FCS experiments. (a) Characteristic diffusion time (correlation time of diffusion τ_D_) (log scale). (b) Autocorrelation curves of FCS experiments performed during aggregation in the presence of HA-RB 100 nM. (c) Contour plot of normalized autocorrelation curves shown in (b).

We finally tested our system with a different method, also based on fluorescence microscopy, that also provides information on the hydrodynamic diameter of diffusing fluorescent aggregates, i.e., fluorescence correlation spectroscopy (FCS).^24,25^ Differently from nanotracking with light-sheet microscopy, though, information from FCS comes as ensemble information, with no details on individual aggregates, which are in turn directly observed and tracked in light-sheet microscopy. FCS, in turn, is extremely sensitive and easy to perform and analyze. We monitored with FCS the aggregation of GAPC1 on a 300 μL well sealed onto a coverglass, in the exact same conditions as other aggregation assays, with [HA-RB] = 100 nM. Concentration of the probe was higher than typical concentration used in FCS (∼1 nM), yet we only monitored the active HA-RB, i.e., the probe bound to protein aggregates, expectedly a much lower fraction. Figure 5b shows the correlograms obtained during time, depicted in three colors to better highlight the three phases of aggregation: preaggregation (green), oligomeric (orange), and particulate phase (red). The correlograms in the preaggregation phase show a low intercept *G*(τ = 0) and inflection point at short correlation times (small τ_D_), suggesting the presence of very small fluorescent objects in relatively high concentration. During the oligomeric phase, the inflection point progressively shifts to longer correlation times, confirming that in this second part of the lag time the average size of oligomeric aggregates starts to increase. This behavior becomes clear also from the fitting of the correlograms to extract the characteristic diffusion time τ_D_, which grows 2–5 times during the oligomeric phase. τ_D_ is linearly proportional to the hydrodynamic diameter and can yield a measure of an average size of the aggregates. Furthermore, also the intercept increases, as expected when many preaggregates coalesce into a lower number of larger oligomeric aggregates. After 40 min, at the beginning of the particulate phase, FCS reveals a huge growth of hydrodynamic diameter and an additional decrease in concentration of these larger aggregates (strong increase in the *G*(τ = 0) intercept). We could also observe a greater than 10-fold increase of τ_D_ (Figure 5a)—and thus of the average hydrodynamic diameter of the aggregates—finally, the very large aggregates grow beyond the measurable range of the FCS technique.

## Discussion

This work aimed to gain control of monitoring the “oligomeric phase” of the redox-triggered aggregation of the AtGAPC1 protein, a step that remains elusive to light scattering-based techniques and that could also not be investigated with fluorescence methods using ThT or Congo Red as fluorescent probes. Based on previous observations,^20^ the persistence of the glutathionylated state across the lag time results in misfolding of AtGAPC1, which causes its irreversible collapse into insoluble aggregates slowly growing from oligomeric ensembles to large, micrometric aggregates. This lag time is therefore a key period that marks the boundary between temporary protection of the catalytic cysteine and irreversible loss of catalytic activity. The events occurring at this stage are difficult to monitor, and it is still unclear whether AtGAPC1 undergoes conformational changes in the preaggregation phase that, without impacting the native form, could result in partial misfolding of the protein and thus be the spark for the aggregation process in the later phases. The oligomeric phase of this redox-triggered aggregation can be regarded as a finely controlled paradigm of the formation of nonfibrillar early aggregates, which are very difficult to observe due to the poor applicability of fibril-specific fluorescent probes such as ThT.

HA-RB has proven to bind with high affinity to protein aggregates, since the early stages of aggregation, while it does not interact with the native (tetrameric) protein. Affinity, in particular, is an important issue for other fluorescents of fluorogenic probes such as ThT, which, due to the low association constant, needs to be employed at rather high concentration (15–50 μM) to push its association with protein aggregates. In contrast, HA-RB can be used at an extremely low concentration of hyaluronan chain (nanomolar range), yielding very low background and laying the basis for high S/N detection.

This probe is made of a biopolymer whose backbone is not intrinsically luminescent but that is made fluorogenic via the functionalization with rhodamine B dyes; such dyes also modulate the solubility properties of hyaluronan, making it slightly hydrophobic, thus (i) pushing the polymer to collapse into nanogels and (ii) making the nanogels highly sensitive to the surface chemistry of protein aggregates. Considering previous knowledge on HA-RB chemistry, in particular, concerning its high-affinity interaction with soft and hydrophobic surfaces such as PEG shell in silica-PEG nanoparticles^26^ or various polymers in micro- and nanoplastics,^23^ its binding to protein aggregates and the ability to discriminate from tetrameric AtGAPC1 protein are likely to occur on partially misfolded protein exposing hydrophobic residues, which then undergoes formation of high order oligomers. The binding event leads to a moderate fluorescence enhancement that is exploited here to monitor early and mature aggregates with a range of fluorescence-based techniques, with special emphasis on the real-time quantification of transient oligomeric species. The observed fluorescence enhancement can be explained with the partial unquenching driven by hydrophobic interactions between HA-RB and the misfolded AtGAPC1 protein: the hydrophobic residues exposed in misfolded proteins and (early) aggregates interact with RB dyes, with a consequent decrease of self-quenching interactions between RB dyes in HA-RB nanogels.

Nonetheless, hydrophobic interactions are only one part of the driving force leading to the observed high affinity of HA-RB toward protein aggregates. The role of polyanionic GAGs in triggering and modulating protein aggregation is widely known, even though the actual mechanism of interaction taking place in vivo between GAGs and proteins, misfolded or in form of oligomers at the early stage of aggregation, has not yet been fully elucidated.^28−30^ Furthermore, the importance of the simultaneous presence of multiple negative charges and of a partially hydrophobic structure has been highlighted in the broad literature on LCOs and LCPs.^14^ For most LCOs, indeed, the mechanism leading to amyloid sensing is a result of interactions attributed to some apolar functional groups (ester groups in particular) that provide sensitivity to the solvation environment and other negatively charged functional groups responsible for selectivity between monomeric and aggregated protein conformations. Restriction of intramolecular motion by binding alone does not seem to be sufficient to explain the signal enhancement, and hydrophobicity-induced unquenching has been identified as the main photophysical mechanism for luminescence enhancement. The comparison with ANS, a molecular probe purely based on the hydrophobic effect, is interesting in this context. ANS, similarly to ThT, labels protein aggregates, but it fails in clearly highlighting the early stages of the aggregation (Figure S7), whereas HA-RB shows the mentioned sudden fluorescence enhancement since the very beginning of the redox-induced aggregation. With HA-RB being a polymer, a cooperative binding may be the reason for this sensitive and early fluorescence enhancement, based on a complex pattern of interactions both with hydrophobic patches and with charged residues of the early aggregates.

The high signal-to-noise ratio emerging from such a low local background emission is the strongest point of HA-RB: the average luminescence enhancement measured in fluorometry appears as not exceptionally high (∼250%), but the accumulation of probes at very low concentration on the aggregates allows for strong accumulation of localized emission, as in other microscopy techniques for super resolution (e.g., BALM^31,32^ or PAINT^33^). In addition, we can presume the real enhancement of PLQY to be higher than ∼250% as suggested by the lifetime analysis, which together with the local accumulation could explain the high S/N observed in microscopy. The high value of this novel probe emerges clearly from results obtained with techniques that need the observation of individual protein aggregates to be tracked (nanotracking with laser-sheet microscopy) or to be correlated to intensity fluctuations in the confocal volume (FCS). In both cases, a clear emission signal from the individual aggregate must stand out from a very dark background. This condition can only be met when the probe has (i) a very high affinity and (ii) a large emission enhancement upon interaction. With these methods, we could gain information on the smallest aggregate size, ranging from 10 to 100 nm, also monitoring each of them individually, possibly opening the door to track the fate of such individual early or oligomeric aggregates in more complex media and conditions.

## Conclusions

In conclusion, we report the application of the fluorogenic HA-RB probe to monitor the aggregation process of plant glycolytic enzyme GAPC1 that occurs under oxidative stress. In particular, we highlighted the peculiar behavior of HA-RB that appears sensitive since the early steps of the aggregation kinetics, showing an onset of fluorescence enhancement where scattering or ThT fluorescence appear silent and only start to increase after the lag phase. These results suggest that HA-RB has a high affinity for early aggregates, and indeed, it could be used even at an extremely low concentration of the hyaluronan chain (100 nM) and still provide clear fluorescence enhancement upon aggregation. The combination of high signal-to-noise provided by the HA-RB fluorogenic probe, its high affinity toward this type of aggregate, and the tailored optical setup allowed us to distinctly track the diffusion of fluorescent aggregates in the size range below the diffraction limit (10–100 nm) and to monitor the evolution of the size distribution in time. Nanotracking with light-sheet microscopy or FCS in a confocal microscope allowed us to follow in real time the aggregation process of the protein showing a size distribution that shifts and broadens toward larger size values as the aggregation proceeds, starting at a size range just above the diameter of the tetrameric protein (∼9 nm) that can be accessed via analysis of diffusion properties.

These results suggest, together with the intrinsic versatility of the reported fluorogenic hyaluronan, that the HA-RB probe could be a valid tool to investigate the elusive lag phase of protein aggregation kinetics even with microscopy and monitoring single aggregates, possibly opening to monitor the fate of these early aggregates in more complex environments.

## Supporting Information Available

Chemicals, experimental protocols and methods, nanotracking analysis, and instrumental details (DLS, microscopes, absorbance, and emission spectrometers) (PDF)

## Abbreviations

RB: Rhodamine B
HA-RB: hyaluronic acid functionalized with rhodamine B
FCS: fluorescence correlation spectroscopy
GAPDH: glyceraldehyde-3-phosphate dehydrogenase
AtGAPC1: cytoplasmic glyceraldehyde-3-phosphate dehydrogenase 1 from *Arabidopsis thaliana*
ThT: thioflavin T
FLIM: fluorescence lifetime imaging microscopy
PLQY: photoluminescence quantum yield
BALM: binding activated localization microscopy
PAINT: point accumulation for imaging in nanoscale topography
LCO: luminescent conjugated oligomer
LCP: luminescent conjugated polymer
GAG: glycosaminoglycan
PEG: polyethylene glycol
S/N: signal-to-noise ratio
NA: numerical aperture
GSH: reduced glutathione
TCEP: tris(2-carboxyethyl)phosphine
