# Supplementary material for: Fluorogenic Hyaluronan Nanogels Track Individual Early Protein Aggregates Originated under Oxidative Stress
Source: ACS Appl Mater Interfaces. 2024 Jan 9;16(3):3056–63. doi: 10.1021/acsami.3c13202 (PMC10811615; doi:10.1021/acsami.3c13202)
Supplement: Supplementary file 1 — am3c13202_si_001.pdf [file am3c13202_si_001.pdf]

SUPPORTING INFORMATION

Fluorogenic hyaluronan nanogels track individual early protein aggregates originated under oxidative stress

*Matteo Cingolani,[a], Francesca Lugli,[a] Mirko Zaffagnini[b] and Damiano Genovese *[a]*

[a]Dipartimento di Chimica "Giacomo Ciamician", Università di Bologna, 40126 Bologna, Italy.

[b]Dipartimento di Farmacia e Biotecnologie, Università di Bologna, 40126 Bologna, Italy

Table of Contents

1) Chemicals

2) Aggregation protocol under controlled oxidative stress

3) DLS

4) Photophysical characterization of HA-RB nanogels

5) Confocal microscopy

6) Light-sheet microscopy

7) Nanotracking Analysis (NTA)

8) Control experiments: aggregation kinetics of AtGAPC1 in absence or in presence of fluorescent probes

1) Chemicals

All reagents and solvents were used as received without further purification: Rhodamine B isothiocyanate (RITC) was purchased from Aldrich. Reagent grade NaCl and phosphate buffer were purchased from Fluka. A MilliQ Millipore system was used for the purification of water (resistivity <18 MΩ). Sodium hyaluronate (HA 200-600 kDa) was purchased from Contipro (Czech Republic).

2) Aggregation protocol under controlled oxidative stress

To test the interaction of the HA-RB with tAtGAPC1 with a spectrofluorimetric-scattering assay, we prepared a 2 mL aggregation solution adding WT (5 μM), HA-RB (100 nM), NAD (0.14 mM) in Tris/EDTA pH=7.4 buffer, split it in two reduced volume cuvettes to monitor fluorescence and scattering respectively. The fluorescence and scattering signals were first monitored for 15 minutes before adding the oxidative stress factors. After that, the solutions are joined again and H_2O_2 (0.125 mM) and GSH (0.625 mM) are added. This solution is immediately split again and both signals have been monitored for 100 minutes when the aggregation kinetic reaches its plateau. All experiments have been performed at controlled temperature of 25 °C. In the experiments involving the tuning of the lag-phase it has also been added to the solution NaCl at a concentration of 200 mM before adding the oxidative stress factors.

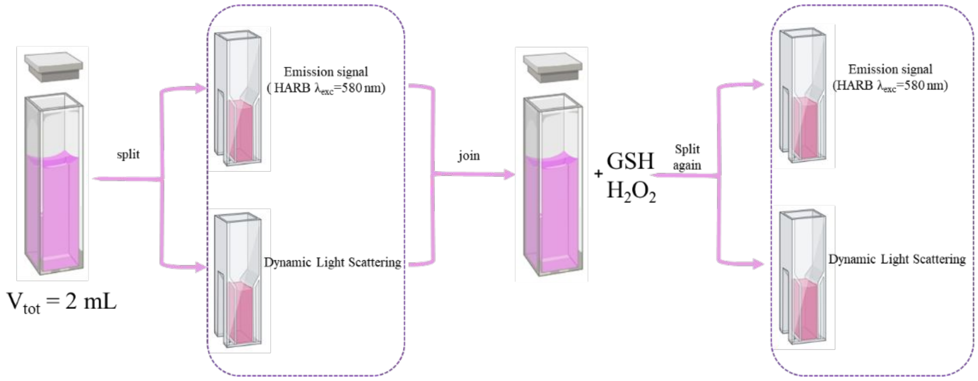

Figure S1. Workflow for cuvette DLS and fluorescence aggregation assays.

3) DLS

The determination of the NP hydrodynamic diameter distributions was carried out through DLS measurements employing a Malvern Nano ZS instrument with a 633 nm laser diode. Samples were housed in disposable polystyrene cuvettes of 1 cm optical path length using water as solvent. The width of DLS hydrodynamic diameter distribution is indicated by PdI (polydispersion index). In the case of a monomodal distribution (Gaussian) calculated by means of cumulant analysis, PdI = $(\sigma/Z_{avg})^2$, where σ is the width of the distribution and Z_{avg} is average diameter of the particles population, respectively.

4) Photophysical characterization of HA-RB nanogels

UV−vis absorption spectra were recorded at 25 °C by means of Perkin-Elmer Lambda 45 spectrophotometer. Quartz cuvettes with optical path length of 1 cm were used. The fluorescence spectra were recorded with an Edinburgh FLS920 equipped with a high speed HS773-04 photomultiplier. The same instrument connected to a PCS900 PC card was used for the TCSPC experiments. Luminescence quantum yields (uncertainty, ±15%) were determined using rhodamine 101 solution in ethanol as a reference (Φ = 1.0). Fluorescence intensities were corrected for inner filter effects according to standard methods.

To evaluate the photophysical properties of the HA-RB probe we acquired absorbance and emission for calculation of photoluminescence quantum yield (PLQY). Rhodamine B solution in ethanol was used as a reference (PLQY=0.65). HA-RB spectra were recorded both in ethanol and water. The calculated photoluminescence quantum yield for the probe in the two different solvent is 0.39 for HA-RB in ethanol and 1.2- $\times 10^{-3}$ for HA-RB in Milli-Q water (Figure S2).

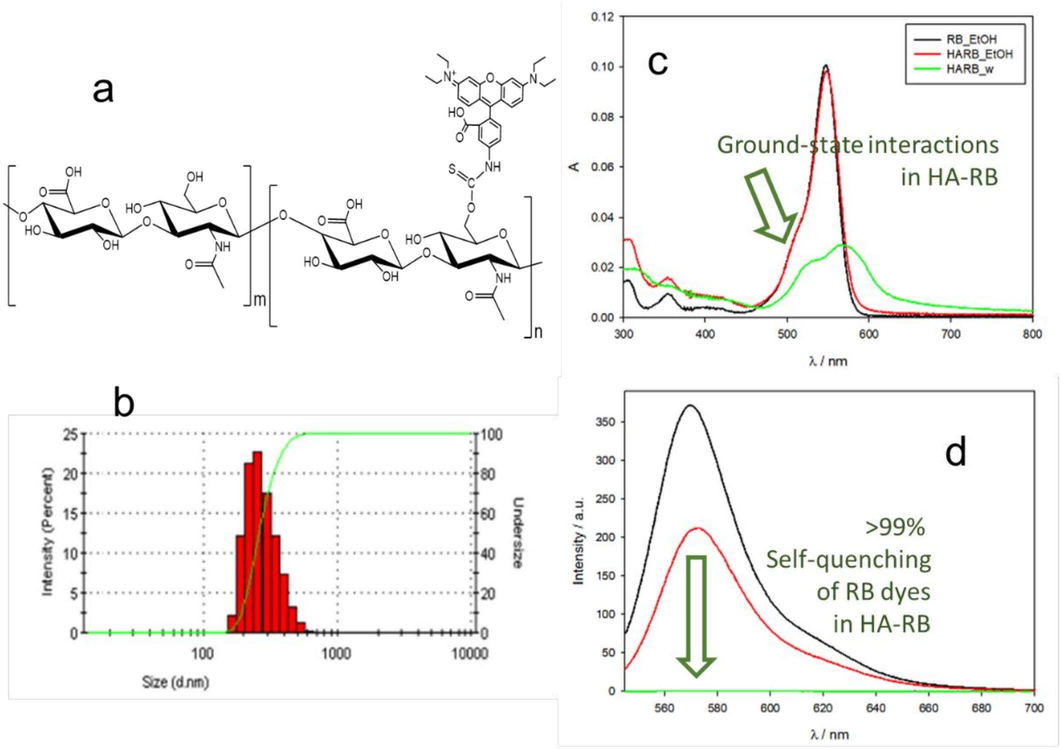

Figure S2. a*) Molecular structure of rhodamine functionalized hyaluronic acid HA-RB. b) Hydrodynamic distribution of HA-RB in water as obtained from Dynamic light scattering measurements (dH = 320 nm, polydispersity index PdI = 0.287). c) Absorbance and d) emission spectra (λ_{exc} = 530 nm) of the reference dye Rhodamine B in ethanol (RB_EtOH) and of HA-RB in ethanol and milliQ water solution (HA-RB_EtOH and HA-RB-w, respectively, both at [HA-RB] = 38 nM).*

5) Confocal microscopy

Confocal images were registered on a Nikon A1R microscope with an oil immersion, high NA objective (NA = 1.45, magnification 100×), an excitation laser at 561 nm and a GaAsP PMT with an emission filter at 595/50 nm.

FCS was acquired with a time-correlated single photon counting (TCSPC) system (PicoQuant GmbH Berlin), using a 405 nm pulsed excitation laser at a 10 MHz repetition rate or a 561 nm CW laser, a 560 nm long-pass emission filter or 600/50 nm bandpass filter, a hybrid PMA detector and a PicoQuant PicoHarp 300 correlation board.

6) Light-sheet microscopy

Laser-sheet wide field for nanoparticle tracking analysis (NTA) setup: a Melles Griot 43 ion tuneable laser (model 35 lap) was used as the excitation source (λ_{exc}= 514 nm) and two plano-convex cylindrical lenses (CL #1 focus distance is 200 mm and CL#2 is 50 mm) as beam shapers in order to have a thin laser-sheet in the optical path of the detector (thickness of laser sheet ca. 10 microns, beam waist ca. 1 mm). An Olympus UPlanFL N 10x Microscope Objective collected the signal from the sample holder, which was then filtered with a long-pass filter (550 nm) and sent to an EMCCD PhotonMAX:512B. All the acquisitions have been performed in *Non-Overlap Mode* with 35 msec exposure time and acquiring 1000 frames (calculated frame per second FPS= 14)

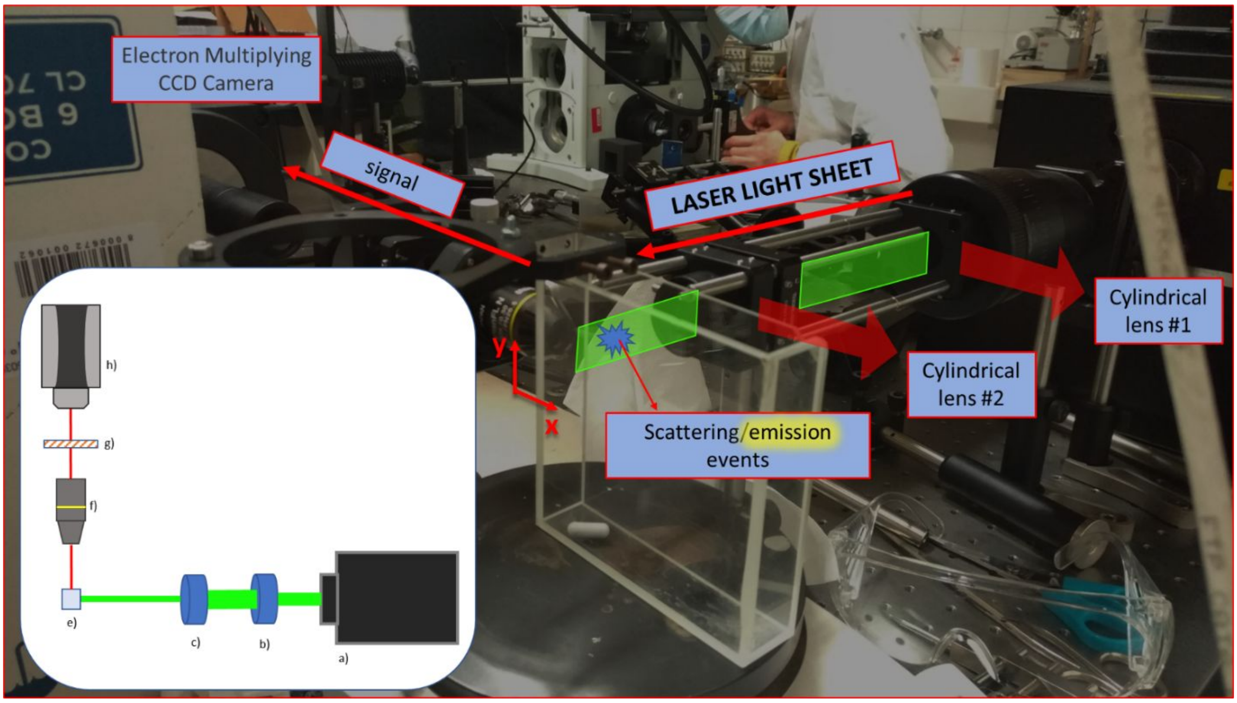

Figure S3. *Picture of the optical setup and inset with the simplified scheme: a) laser source; b) cylindrical lens 1; c) cylindrical lens 2; d) cuvette location; e) objective; f) emission filter; g) detection camera*

7) Nanotracking Analysis (NTA)

Effective image size has been calibrated acquiring a picture of a PELCO FORMVAR 400 mesh. Calibration has been performed measuring 10 holes and dividing it by the pitch value. Calculated pixel size is 1.15 µm.

The homemade laser-sheet wide-field optical setup has been calibrated for Nanotracking using highly monodispersed polystyrene (PS) nanoparticles previously synthesized and available in our lab, having three different hydrodynamic diameters (90 nm, 150 nm, 170 nm indicated respectively as PS 1, PS 2 and PS 3). Each sample was diluted in a reduced volume quartz cuvette (500 µL) in a thermalised bath at 25 °C. For each sample 1000 frames with exposure time of 35 msec (14 FPS) have been acquired. After that, each acquisition has been analysed with the NanotrackJ plugin of ImageJ programme. In order to evaluate the analysis algorithm both the available methods – Walker's and Default Methods – have been used for the distribution fitting. These results have been then compared to DLS measurement of the same cuvettes with very good agreement of the fitting data (fig xx).

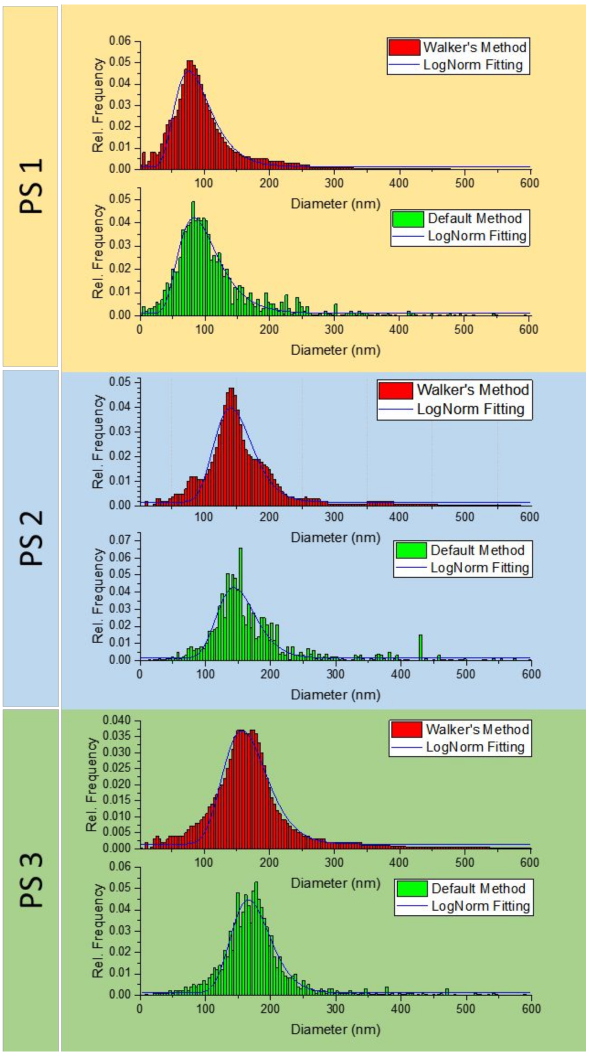

Figure S4 *– Dimensional distribution of the population of PS nanoparticles. The diameter values of the NTA analysis are in good agreement with the values in the table obtained with a DLS measurement (Table S1).*

	DLS		NanoTracking (Laser Sheet)	
samples	dH	PDI	Walker Method	Standard Method
PS_1	80	0.107	87	94
PS_2	159	0.059	147	149
PS_3	179	0.123	165	172

Table S1. Comparison between DLS and nanotracking results for the standard samples of polystyrene nanoparticles.

8) **Control experiments:** aggregation kinetics of AtGAPC1 in absence or in presence of fluorescent probes.

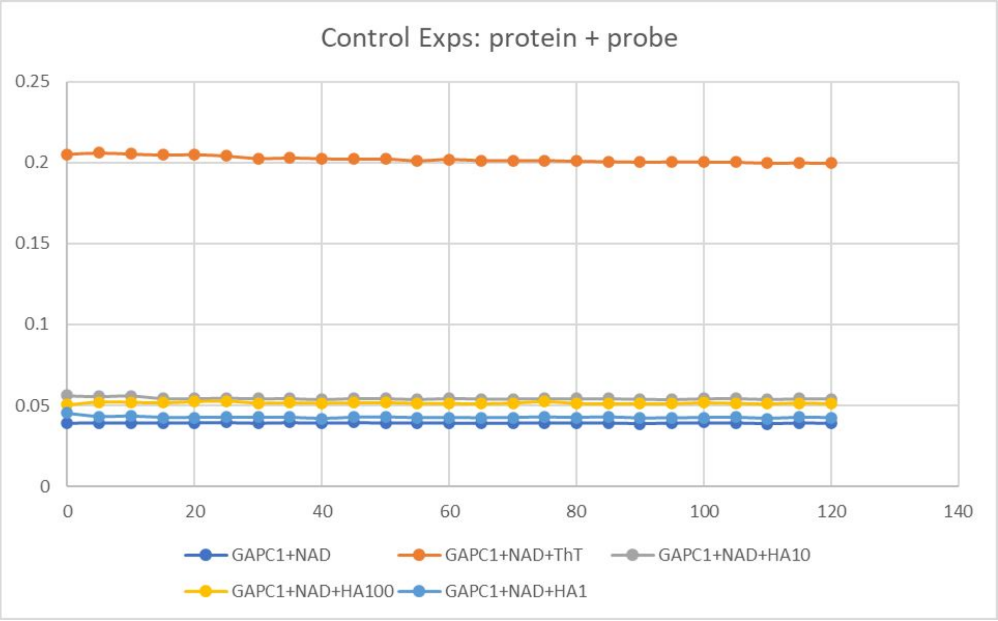

Figure S5 – Control experiments of aggregation, measured via turbidity (absorbance at 400 nm) in absence or in presence of fluorescent probes ThT (25 µM) or HA-RB (10, 100 or 1000 nM), here in absence of the redox trigger that initiates the aggregation process (H_2O_2 and GSH).

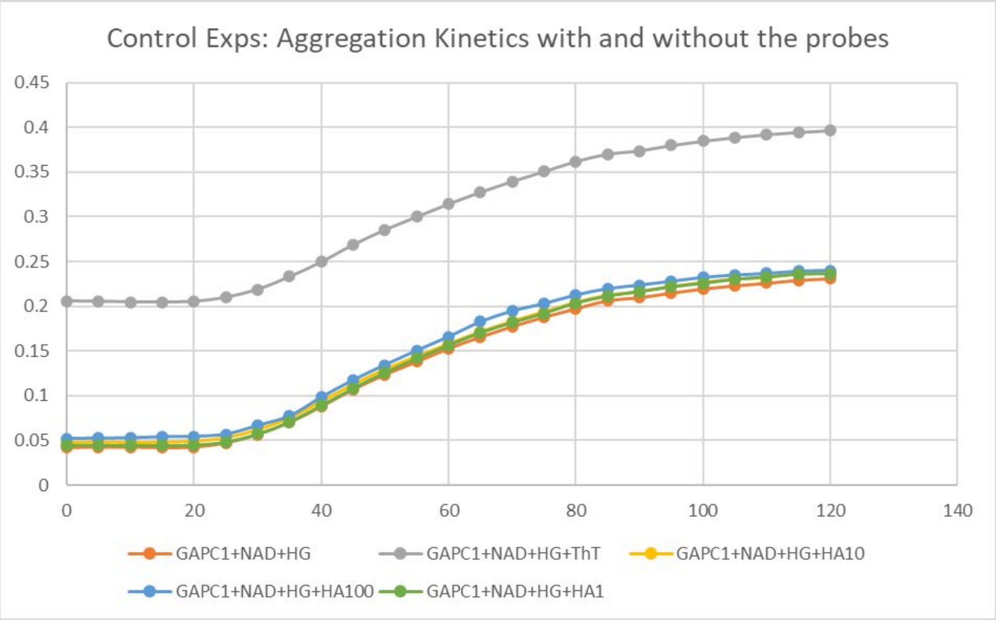

Figure S6 – Control experiments of aggregation of AtGAPC1, measured via turbidity (absorbance at 400 nm) in absence or in presence of fluorescent probes ThT (25 μM) or HA-RB (10, 100 or 1000 nM), here in presence of the redox trigger that initiates the aggregation process (H_2O_2 and GSH). Absorbance at 400 nm of sample including ThT is higher because of the absorption by ThT, yet the trend does not vary. Aggregation trends do not vary with and without fluorescent probes, at all concentrations of HA-RB investigated.

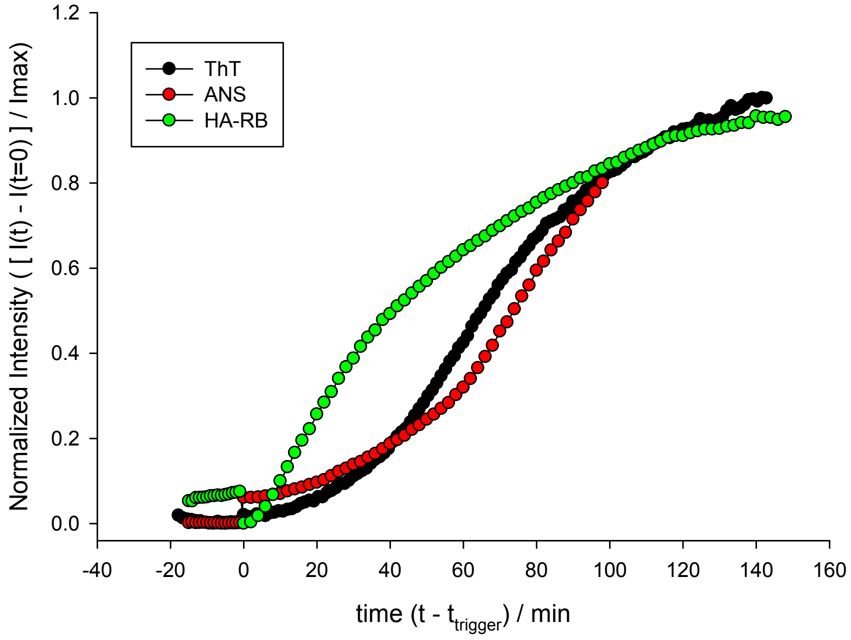

Figure S7 – Comparison of three different fluorescent probes: ThT (black circles), ANS (red circles) and HA-RB (green circles). Trends have been normalized to better compare the onset of

fluorescence enhancement in the early steps of aggregation. Original enhancement factors for ThT, ANS and HA-RB were 4.1, 7.3 and 2.4, respectively.
